# Supplementary material for: Possible Role of miR-375-3p in Cervical Lymph Node Metastasis of Oral Squamous Cell Carcinoma
Source: Cancers (Basel). 2024 Apr 13;16(8):1492. doi: 10.3390/cancers16081492 (PMC11049256; doi:10.3390/cancers16081492)
Supplement: Supplementary file 1 [file cancers-16-01492-s001.zip › Table S1.pdf]

Table S1. Clinicopathological parameters of patients with oral squamous cell carcinoma in this study.

Parameter	No recurrence (n=15)	Nodal relapse (n=15)	*P*-value
Age			0.4889
Median (Range)	60 (48-91)	71 (37-88)	
Gender			0.4642
Male	9	7	
Female	6	8	
Primary site			
Tongue	15	15	
Differentiation			0.0393
G 1	11	6	
G 2	4	4	
G 3	0	5	
Clinical stage			0.2733
Stage Ⅰ	9	6	
Stage Ⅱ	6	9	
