# Supplementary material for: Possible Role of miR-375-3p in Cervical Lymph Node Metastasis of Oral Squamous Cell Carcinoma
Source: Cancers (Basel). 2024 Apr 13;16(8):1492. doi: 10.3390/cancers16081492 (PMC11049256; doi:10.3390/cancers16081492)
Supplement: Supplementary file 1 [file cancers-16-01492-s001.zip › Table S2.pdf]

Table S2. ON-TARGETplus small interfering RNAs specific for miR-375-3p target genes.

Gene symbol	Gene name	Catalog number
QKI	Quaking homolog, KH domain RNA binding	L-024905-01
MTDH	Metadherin	L-018531-01
YAP1	Yes1 Associated Transcriptional Regulator	L-012200-00
CBX3	Chromobox homolog 3	L-010033-00
CHSY1	Chondroitin sulfate synthase 1	L-021339-01
MAPKAP1	Mitogen-activated protein kinase associated protein 1	L-014315-00
LDHB	lactate dehydrogenase B	L-009779-00
UBE2E2	ubiquitin-conjugating enzyme E2E 2	L-031782-00
USP1	ubiquitin specific peptidase 1	L-006061-00
C10orf26	Chromosome 10 open reading frame 26	L-019421-02
LSM12	LSM12 homolog	L-015641-01
PLEKHA3	pleckstrin homology domain containing, family A member 3	L-004319-01
EBPL	emopamil binding protein-like	L-014920-01
OGFOD1	2-oxoglutarate and iron-dependent oxygenase domain containing 1	L-021197-01
CORO2A	coronin, actin binding protein, 2A	L-011420-00
PTPMT1	protein tyrosine phosphatase, mitochondrial 1	L-029988-01
CSTF2	cleavage stimulation factor, 3' pre-RNA, subunit 2, 64kDa	L-011246-00
POC1B	POC1 centriolar protein homolog B	L-015667-01
EIF4G3	eukaryotic translation initiation factor 4 gamma, 3	L-019530-00
PPPDE2	PPPDE peptidase domain containing 2	L-024576-02
C1QBP	complement component 1, q subcomponent binding protein	L-011225-01
RPN1	ribophorin I	L-018903-01
MBD2	methyl-CpG binding domain protein 2	L-011555-00
DCUN1D4	DCN1, defective in cullin neddylation 1, domain containing 4	L-014118-01
TMEM55A	transmembrane protein 55A	L-013808-01
ARL4C	ADP-ribosylation factor-like 4C	L-012398-00
PRDX1	peroxiredoxin 1	L-010338-00
KIAA1524	Cellular Inhibitor of PP2A	L-014135-01
MOBKL1B	MOB1, Mps One Binder kinase activator-like 1B	L-021097-00
CNN3	calponin 3, acidic	L-011612-02

ASF1A	ASF1 anti-silencing function 1 homolog A	L-020222-02
PDGFC	platelet derived growth factor C	L-013154-01
PAQR3	progestin and adipoQ receptor family member III	L-008053-00
CEPT1	choline/ethanolamine phosphotransferase 1	L-010350-01
TIMM8A	translocase of inner mitochondrial membrane 8 homolog A	L-010342-00
DEK	DEK oncogene	L-003881-00
ATPAF1	ATP synthase mitochondrial F1 complex assembly factor 1	L-013738-01
