# Supplementary material for: Possible Role of miR-375-3p in Cervical Lymph Node Metastasis of Oral Squamous Cell Carcinoma
Source: Cancers (Basel). 2024 Apr 13;16(8):1492. doi: 10.3390/cancers16081492 (PMC11049256; doi:10.3390/cancers16081492)
Supplement: Supplementary file 1 [file cancers-16-01492-s001.zip › Table S3.pdf]

**Table S3**. Upregulated microRNAs in primary oral squamous cell carcinoma tissues.

| microRNA | Fold change | *P*-value |
|---|---|---|
| hsa-miR-424-3p | 9.76 | 8.09E-09 |
| hsa-miR-21-3p | 7.85 | 8.09E-09 |
| hsa-miR-21-5p | 5.33 | 2.21E-13 |
| hsa-miR-31-3p | 4.20 | 3.53E-02 |
| hsa-miR-503-5p | 4.14 | 8.69E-09 |
| hsa-miR-196a-5p | 3.65 | 2.64E-02 |
| hsa-miR-31-5p | 3.62 | 1.27E-02 |
| hsa-miR-146b-5p | 3.40 | 2.51E-04 |
| hsa-miR-455-5p | 2.87 | 3.39E-02 |
| hsa-miR-424-5p | 2.83 | 1.61E-02 |
| hsa-miR-1290 | 2.63 | 4.70E-02 |
| hsa-miR-223-3p | 2.61 | 8.81E-03 |
| hsa-miR-212-3p | 2.59 | 2.31E-02 |
| hsa-miR-542-5p | 2.58 | 8.24E-03 |
| hsa-miR-25-5p | 2.47 | 3.79E-02 |
| hsa-miR-181d-5p | 2.40 | 1.80E-02 |
| hsa-miR-642a-3p | 2.38 | 9.54E-03 |
| hsa-miR-421 | 2.37 | 2.87E-02 |
| hsa-miR-431-5p | 2.36 | 4.19E-02 |
| hsa-miR-1260a | 2.35 | 2.91E-02 |
| hsa-miR-409-3p | 2.23 | 3.32E-02 |
| hsa-miR-34c-3p | 2.22 | 9.54E-03 |
| hsa-miR-455-3p | 2.19 | 3.31E-03 |
| hsa-miR-181b-5p | 2.16 | 1.83E-03 |
| hsa-miR-130b-3p | 2.13 | 8.33E-03 |
| hsa-miR-3663-3p | 2.09 | 3.31E-03 |
| hsa-miR-4449 | 2.07 | 3.11E-02 |
| hsa-miR-1246 | 2.06 | 7.39E-03 |
| hsa-miR-4690-5p | 2.06 | 7.76E-03 |
| hsa-miR-766-3p | 2.06 | 3.11E-02 |
| hsa-miR-181a-3p | 2.04 | 1.61E-02 |
| hsa-miR-4306 | 2.04 | 3.11E-02 |
| hsa-miR-485-3p | 2.01 | 3.11E-02 |
