# Supplementary material for: Possible Role of miR-375-3p in Cervical Lymph Node Metastasis of Oral Squamous Cell Carcinoma
Source: Cancers (Basel). 2024 Apr 13;16(8):1492. doi: 10.3390/cancers16081492 (PMC11049256; doi:10.3390/cancers16081492)
Supplement: Supplementary file 1 [file cancers-16-01492-s001.zip › Table S4.pdf]

Table S4. Downregulated microRNAs in primary oral squamous cell carcinoma tissues.

microRNA	Fold change	*P*-value
hsa-miR-375	-53.98	1.62E-08
hsa-miR-5580-3p	-38.65	1.59E-14
hsa-miR-617	-16.72	6.23E-20
hsa-miR-6746-5p	-15.25	5.63E-06
hsa-miR-451a	-10.03	2.86E-03
hsa-miR-3935	-8.41	1.48E-07
hsa-miR-1224-5p	-8.33	2.41E-06
hsa-miR-4462	-7.87	4.76E-07
hsa-miR-6875-5p	-5.49	7.11E-05
hsa-miR-6510-3p	-5.31	2.78E-11
hsa-miR-6504-3p	-3.84	1.02E-05
hsa-miR-6801-5p	-3.83	2.64E-02
hsa-miR-99a-5p	-3.81	7.47E-05
hsa-miR-6808-5p	-3.73	1.01E-04
hsa-miR-149-5p	-3.62	2.98E-02
hsa-miR-6762-5p	-3.48	7.39E-03
hsa-miR-125b-2-3p	-3.32	6.47E-03
hsa-miR-486-5p	-3.31	6.40E-03
hsa-miR-4521	-3.16	4.17E-02
hsa-miR-6851-5p	-3.03	5.07E-04
hsa-miR-30a-5p	-2.78	5.53E-04
hsa-miR-101-3p	-2.76	5.63E-06
hsa-miR-6756-5p	-2.59	1.41E-05
hsa-miR-365b-5p	-2.57	3.59E-02
hsa-miR-376c-3p	-2.53	2.93E-02
hsa-miR-6086	-2.49	8.16E-05
hsa-miR-30a-3p	-2.46	2.64E-02
hsa-miR-411-5p	-2.41	1.80E-02
hsa-miR-100-5p	-2.31	1.10E-02
hsa-miR-203b-3p	-2.29	3.41E-03
hsa-miR-3065-5p	-2.26	2.86E-03
hsa-miR-4732-5p	-2.19	2.64E-02
hsa-miR-6777-3p	-2.18	2.73E-05
hsa-miR-4717-5p	-2.17	3.65E-04

hsa-miR-139-5p	-2.15	4.97E-03
hsa-miR-99a-3p	-2.15	1.08E-05
hsa-miR-27b-3p	-2.13	5.52E-03
hsa-miR-378c	-2.12	7.43E-03
hsa-miR-3911	-2.11	1.62E-02
hsa-miR-3065-3p	-2.11	2.50E-03
hsa-miR-6880-5p	-2.10	1.15E-02
hsa-miR-497-5p	-2.08	3.35E-02
hsa-miR-422a	-2.05	3.31E-03
hsa-miR-654-3p	-2.05	4.78E-02
hsa-miR-3613-5p	-2.04	3.11E-02
hsa-miR-3194-5p	-2.04	2.23E-02
hsa-miR-363-3p	-2.04	3.39E-02
hsa-miR-195-5p	-2.03	1.15E-02
hsa-miR-664a-5p	-2.01	3.35E-02
