# Supplementary material for: Possible Role of miR-375-3p in Cervical Lymph Node Metastasis of Oral Squamous Cell Carcinoma
Source: Cancers (Basel). 2024 Apr 13;16(8):1492. doi: 10.3390/cancers16081492 (PMC11049256; doi:10.3390/cancers16081492)
Supplement: Supplementary file 1 [file cancers-16-01492-s001.zip › Table S5.pdf]

Table S5. Genes regulated by miR375-3p in human oral squamous cell carcinoma cells.

Gene symbol	Gene name	Fold change	*P*-value
QKI	quaking homolog, KH domain RNA binding	-9.93	0.0114
SUB1	SUB1 homolog	-7.83	0.0105
PSIP1	PC4 and SFRS1 interacting protein 1	-7.53	0.0115
ASPH	aspartate beta-hydroxylase	-6.57	0.0114
MTDH	metadherin	-6.55	0.0202
MAT2B	methionine adenosyltransferase II, beta	-6.48	0.0105
YAP1	Yes-associated protein 1	-5.54	0.0114
TBC1D5	TBC1 domain family, member 5	-5.32	0.0114
PGM2	phosphoglucomutase 2	-5.16	0.0114
WWC2	WW and C2 domain containing 2	-5.00	0.0113
CBX3	chromobox homolog 3	-4.91	0.0119
CHSY1	chondroitin sulfate synthase 1	-4.84	0.0105
ELOVL6	ELOVL family member 6, elongation of long chain fatty acids	-4.83	0.0136
EIF2S1	eukaryotic translation initiation factor 2, subunit 1 alpha, 35kDa	-4.77	0.0105
CLVS1	clavesin 1	-4.74	0.0113
MAPKAP1	mitogen-activated protein kinase associated protein 1	-4.51	0.0140
HDHD1A	haloacid dehalogenase-like hydrolase domain containing 1A	-4.47	0.0114
LDHB	lactate dehydrogenase B	-4.24	0.0142
UBE2E2	ubiquitin-conjugating enzyme E2E 2	-4.17	0.0105
SGMS2	sphingomyelin synthase 2	-4.15	0.0136
ACSL3	acyl-CoA synthetase long-chain family member 3	-4.11	0.0105
SEC23A	Sec23 homolog A	-4.08	0.0114
TMX3	thioredoxin-related transmembrane protein 3	-4.06	0.0122
FAM36A	family with sequence similarity 36, member A	-4.00	0.0150
EIF5A2	eukaryotic translation initiation factor 5A2	-3.99	0.0181
NT5E	5'-nucleotidase, ecto	-3.95	0.0187
CHST11	carbohydrate sulfotransferase 11	-3.87	0.0115
ME1	malic enzyme 1, NADP(+)-dependent, cytosolic	-3.87	0.0153
THBS1	thrombospondin 1	-3.85	0.0358

YWHAZ	tyrosine 3-monooxygenase/tryptophan 5-monooxygenase activation protein, zeta polypeptide	-3.85	0.0144
GOLGA4	golgin A4	-3.84	0.0114
USP1	ubiquitin specific peptidase 1	-3.81	0.0226
RBPJ	recombination signal binding protein for immunoglobulin kappa J region	-3.80	0.0114
SEMA3C	sema domain, immunoglobulin domain, short basic domain, secreted, 3C	-3.78	0.0191
MAL2	mal, T-cell differentiation protein 2	-3.73	0.0114
C10orf26	chromosome 10 open reading frame 26	-3.72	0.0114
MPP5	membrane protein, palmitoylated 5	-3.67	0.0191
LSM12	LSM12 homolog	-3.57	0.0119
PLEKHA3	pleckstrin homology domain containing, family A member 3	-3.48	0.0105
CDCA7L	cell division cycle associated 7-like	-3.48	0.0237
RCN2	reticulocalbin 2, EF-hand calcium binding domain	-3.45	0.0181
EBPL	emopamil binding protein-like	-3.42	0.0142
BCL10	B-cell CLL/lymphoma 10	-3.38	0.0113
MLEC	malectin	-3.35	0.0234
SKP2	S-phase kinase-associated protein 2	-3.32	0.0224
TH1L	TH1-like	-3.31	0.0229
EFTUD2	elongation factor Tu GTP binding domain containing 2	-3.30	0.0181
TTC7B	tetratricopeptide repeat domain 7B	-3.29	0.0153
DPY19L1	dpy-19-like 1	-3.23	0.0147
CRIM1	cysteine rich transmembrane BMP regulator 1	-3.21	0.0276
OGFOD1	2-oxoglutarate and iron-dependent oxygenase domain containing 1	-3.18	0.0114
REEP3	receptor accessory protein 3	-3.18	0.0153
FST	follistatin	-3.17	0.0463
FGFR1OP2	FGFR1 oncogene partner 2	-3.17	0.0114
KRT6A	keratin 6A	-3.17	0.0294
CCDC88A	coiled-coil domain containing 88A	-3.17	0.0181
NIN	ninein	-3.15	0.0129

ASAP2	ArfGAP with SH3 domain, ankyrin repeat and PH domain 2	-3.13	0.0113
BIRC5	baculoviral IAP repeat-containing 5	-3.12	0.0207
C3orf63	chromosome 3 open reading frame 63	-3.11	0.0140
PHTF2	putative homeodomain transcription factor 2	-3.10	0.0285
C14orf149	chromosome 14 open reading frame 149	-3.10	0.0113
CORO1C	coronin, actin binding protein, 1C	-3.10	0.0150
USP10	ubiquitin specific peptidase 10	-3.08	0.0211
CORO2A	coronin, actin binding protein, 2A	-3.08	0.0245
SAMD9	sterile alpha motif domain containing 9	-3.07	0.0123
TMEM194A	transmembrane protein 194A	-3.02	0.0328
GOLIM4	golgi integral membrane protein 4	-3.00	0.0231
PGRMC1	progesterone receptor membrane component 1	-2.98	0.0147
PTPMT1	protein tyrosine phosphatase, mitochondrial 1	-2.93	0.0181
TMEM33	transmembrane protein 33	-2.93	0.0105
CSTF2	cleavage stimulation factor, 3' pre-RNA, subunit 2, 64kDa	-2.92	0.0156
TYMS	thymidylate synthetase	-2.91	0.0378
SETD7	SET domain containing 7	-2.90	0.0136
SPAG9	sperm associated antigen 9	-2.90	0.0113
TRIM6	tripartite motif-containing 6	-2.90	0.0214
CENPF	centromere protein F, 350/400ka	-2.89	0.0298
RSU1	Ras suppressor protein 1	-2.89	0.0114
POC1B	POC1 centriolar protein homolog B	-2.89	0.0122
EBAG9	estrogen receptor binding site associated, antigen, 9	-2.88	0.0129
FAM3C	family with sequence similarity 3, member C	-2.88	0.0219
EIF4G3	eukaryotic translation initiation factor 4 gamma, 3	-2.88	0.0105
KPNA4	karyopherin alpha 4	-2.87	0.0168
PTPN12	protein tyrosine phosphatase, non-receptor type 12	-2.85	0.0117
ENAH	enabled homolog	-2.82	0.0224
CNN2	calponin 2	-2.82	0.0265
SYNJ2BP	synaptojanin 2 binding protein	-2.80	0.0105

CHPT1	choline phosphotransferase 1	-2.78	0.0114
XPR1	xenotropic and polytropic retrovirus receptor 1	-2.76	0.0126
PPPDE2	PPPDE peptidase domain containing 2	-2.76	0.0144
UBE3A	ubiquitin protein ligase E3A	-2.76	0.0144
RASSF8	Ras association domain family member 8	-2.75	0.0113
SAR1A	SAR1 homolog A	-2.74	0.0114
C12orf29	chromosome 12 open reading frame 29	-2.74	0.0173
TMEM19	transmembrane protein 19	-2.74	0.0224
SKA2	spindle and kinetochore associated complex subunit 2	-2.73	0.0219
C13orf37	chromosome 13 open reading frame 37	-2.73	0.0274
ARHGAP18	Rho GTPase activating protein 18	-2.73	0.0123
OPA1	optic atrophy 1	-2.72	0.0114
KIAA1191	KIAA1191	-2.72	0.0105
TPR	translocated promoter region	-2.70	0.0153
CDR2	cerebellar degeneration-related protein 2, 62kDa	-2.70	0.0159
COPS8	COP9 constitutive photomorphogenic homolog subunit 8	-2.70	0.0142
C1QBP	complement component 1, q subcomponent binding protein	-2.68	0.0179
RPN1	ribophorin I	-2.67	0.0286
CCDC91	coiled-coil domain containing 91	-2.67	0.0115
LMAN1	lectin, mannose-binding, 1	-2.66	0.0243
ITGA6	integrin, alpha 6	-2.65	0.0140
NUS1	nuclear undecaprenyl pyrophosphate synthase 1 homolog	-2.65	0.0232
FGFBP1	fibroblast growth factor binding protein 1	-2.65	0.0444
WTAP	Wilms tumor 1 associated protein	-2.64	0.0294
MBD2	methyl-CpG binding domain protein 2	-2.63	0.0134
GFM2	G elongation factor, mitochondrial 2	-2.63	0.0153
TIMM17A	translocase of inner mitochondrial membrane 17 homolog A	-2.62	0.0150
USP46	ubiquitin specific peptidase 46	-2.62	0.0343
POLR3G	polymerase III polypeptide G	-2.62	0.0114

SP100	SP100 nuclear antigen	-2.62	0.0114
DDX18	DEAD box polypeptide 18	-2.61	0.0179
DAZAP2	DAZ associated protein 2	-2.60	0.0114
MYBL1	v-myb myeloblastosis viral oncogene homolog-like 1	-2.58	0.0334
KCTD10	potassium channel tetramerisation domain containing 10	-2.56	0.0140
PODXL	podocalyxin-like	-2.55	0.0296
DCUN1D4	DCN1, defective in cullin neddylation 1, domain containing 4	-2.55	0.0142
DIS3	DIS3 mitotic control homolog	-2.52	0.0114
DST	dystonin	-2.51	0.0156
ENOPH1	enolase-phosphatase 1	-2.51	0.0144
MYH10	myosin, heavy chain 10, non-muscle	-2.51	0.0169
C1orf96	chromosome 1 open reading frame 96	-2.51	0.0114
NETO2	neuropilin and tolloid-like 2	-2.48	0.0219
PPP1R2	protein phosphatase 1, regulatory subunit 2	-2.48	0.0130
PEBP1	phosphatidylethanolamine binding protein 1	-2.47	0.0193
SLC35B4	solute carrier family 35, member B4	-2.47	0.0185
WTAP	Wilms tumor 1 associated protein	-2.45	0.0304
TMEM55A	transmembrane protein 55A	-2.45	0.0114
SYNCRIP	synaptotagmin binding, cytoplasmic RNA interacting protein	-2.44	0.0191
NF1	neurofibromin 1	-2.43	0.0125
SMYD2	SET and MYND domain containing 2	-2.42	0.0117
TCF12	transcription factor 12	-2.41	0.0194
AMMECR1	Alport syndrome, mental retardation, midface hypoplasia and elliptocytosis chromosomal region gene 1	-2.41	0.0269
SH3D19	SH3 domain containing 19	-2.41	0.0267
MRPL19	mitochondrial ribosomal protein L19	-2.41	0.0114
SLC25A40	solute carrier family 25, member 40	-2.40	0.0150
ARL4C	ADP-ribosylation factor-like 4C	-2.40	0.0114
BNC1	basonuclin 1	-2.38	0.0117
RER1	RER1 retention in endoplasmic reticulum 1 homolog	-2.37	0.0122

SGOL2	shugoshin-like 2	-2.37	0.0262
PTP4A2	protein tyrosine phosphatase type IVA, member 2	-2.37	0.0165
WWTR1	WW domain containing transcription regulator 1	-2.37	0.0156
INSIG2	insulin induced gene 2	-2.36	0.0150
UNC13B	unc-13 homolog B	-2.36	0.0169
UHRF1	ubiquitin-like with PHD and ring finger domains 1	-2.36	0.0445
GPD1L	glycerol-3-phosphate dehydrogenase 1-like	-2.36	0.0181
SNX6	sorting nexin 6	-2.35	0.0196
EEA1	early endosome antigen 1	-2.35	0.0144
TUBGCP3	tubulin, gamma complex associated protein 3	-2.34	0.0237
OTUD6B	OTU domain containing 6B	-2.33	0.0294
CTBP2	C-terminal binding protein 2	-2.33	0.0105
GCSH	glycine cleavage system protein H	-2.33	0.0456
FAM63B	family with sequence similarity 63, member B	-2.32	0.0182
TAOK3	TAO kinase 3	-2.32	0.0113
FRMD6	FERM domain containing 6	-2.31	0.0140
DERL1	Der1-like domain family, member 1	-2.31	0.0122
KLHL7	kelch-like 7	-2.30	0.0182
AK3L1	adenylate kinase 3-like 1	-2.30	0.0229
PRDX1	peroxiredoxin 1	-2.30	0.0144
EMB	embigin homolog	-2.30	0.0156
CLTB	clathrin, light chain B	-2.29	0.0341
RAB21	RAB21, member RAS oncogene family	-2.29	0.0114
OSBPL8	oxysterol binding protein-like 8	-2.29	0.0274
AIM1	absent in melanoma 1	-2.28	0.0125
PRIM2	primase, DNA, polypeptide 2	-2.28	0.0217
KDSR	3-ketodihydrosphingosine reductase	-2.28	0.0105
MAP2K4	mitogen-activated protein kinase kinase 4	-2.27	0.0219
VGLL4	vestigial like 4	-2.27	0.0130
BAG4	BCL2-associated athanogene 4	-2.26	0.0142
RRP1B	ribosomal RNA processing 1 homolog B	-2.25	0.0147
RAPGEF1	Rap guanine nucleotide exchange factor 1	-2.25	0.0145
KIAA1524	KIAA1524	-2.25	0.0344

SERTAD2	SERTA domain containing 2	-2.25	0.0114
USP31	ubiquitin specific peptidase 31	-2.24	0.0118
WDR1	WD repeat domain 1	-2.24	0.0166
CHIC1	cysteine-rich hydrophobic domain 1	-2.24	0.0117
MBNL1	muscleblind-like	-2.23	0.0114
PRDX3	peroxiredoxin 3	-2.23	0.0243
TMEFF1	transmembrane protein with EGF-like and two follistatin-like domains 1	-2.23	0.0401
AREG	amphiregulin	-2.22	0.0175
ACSL4	acyl-CoA synthetase long-chain family member 4	-2.22	0.0114
CTPS	CTP synthase	-2.22	0.0140
NOM1	nucleolar protein with MIF4G domain 1	-2.21	0.0114
MOBKL1B	MOB1, Mps One Binder kinase activator-like 1B	-2.21	0.0114
BBX	bobby sox homolog	-2.20	0.0180
SET	SET nuclear oncogene	-2.20	0.0165
PRRG1	proline rich Gla 1	-2.19	0.0439
HRSP12	heat-responsive protein 12	-2.19	0.0339
HELLS	helicase, lymphoid-specific	-2.19	0.0322
PAFAH1B1	platelet-activating factor acetylhydrolase 1b, regulatory subunit 1	-2.19	0.0154
LANCL1	LanC lantibiotic synthetase component C-like 1	-2.19	0.0113
CNN3	calponin 3, acidic	-2.19	0.0344
FAM82A2	family with sequence similarity 82, member A2	-2.19	0.0149
SNRPA1	small nuclear ribonucleoprotein polypeptide A'	-2.18	0.0340
C13orf34	chromosome 13 open reading frame 34	-2.18	0.0439
SUZ12	suppressor of zeste 12 homolog	-2.18	0.0354
CFL2	cofilin 2	-2.18	0.0415
MTPN	myotrophin	-2.18	0.0140
NUDT21	nudix-type motif 21	-2.18	0.0158
SNX25	sorting nexin 25	-2.17	0.0144
ZNF828	zinc finger protein 828	-2.17	0.0125
EIF3J	eukaryotic translation initiation factor 3, subunit J	-2.17	0.0122

OXNAD1	oxidoreductase NAD-binding domain containing 1	-2.17	0.0105
AEBP2	AE binding protein 2	-2.16	0.0158
AHCYL1	adenosylhomocysteinase-like 1	-2.15	0.0296
ITSN1	intersectin 1	-2.15	0.0277
UGCG	UDP-glucose ceramide glucosyltransferase	-2.15	0.0147
LAMC2	laminin, gamma 2	-2.14	0.0156
CLTC	clathrin, heavy chain	-2.14	0.0114
MAP2K4	mitogen-activated protein kinase kinase 4	-2.14	0.0181
C15orf52	chromosome 15 open reading frame 52	-2.14	0.0126
EXO1	exonuclease 1	-2.14	0.0275
ASF1A	ASF1 anti-silencing function 1 homolog A	-2.13	0.0250
CHCHD4	coiled-coil-helix-coiled-coil-helix domain containing 4	-2.12	0.0140
GUF1	GUF1 GTPase homolog	-2.12	0.0322
PDGFC	platelet derived growth factor C	-2.12	0.0114
ADSS	adenylosuccinate synthase	-2.11	0.0105
NUP54	nucleoporin 54kDa	-2.11	0.0276
TFAM	transcription factor A, mitochondrial	-2.11	0.0180
SPCS3	signal peptidase complex subunit 3 homolog	-2.11	0.0243
DR1	down-regulator of transcription 1, TBP-binding	-2.11	0.0189
B3GALNT1	beta-1,3-N-acetylgalactosaminyltransferase 1	-2.10	0.0398
C6orf182	chromosome 6 open reading frame 182	-2.10	0.0105
CAV1	caveolin 1, caveolae protein, 22kDa	-2.10	0.0210
TMED5	transmembrane emp24 protein transport domain containing 5	-2.10	0.0178
SH3GLB1	SH3-domain GRB2-like endophilin B1	-2.10	0.0114
PAQR3	progestin and adipoQ receptor family member III	-2.10	0.0113
SH3PXD2A	SH3 and PX domains 2A	-2.10	0.0114
KCTD12	potassium channel tetramerisation domain containing 12	-2.09	0.0284
NLK	nemo-like kinase	-2.09	0.0166
UBLCP1	ubiquitin-like domain containing CTD phosphatase 1	-2.09	0.0142
CEPT1	choline/ethanolamine phosphotransferase 1	-2.08	0.0262

ITGB4	integrin, beta 4	-2.07	0.0193
NUMB	numb homolog	-2.07	0.0122
PDHA1	pyruvate dehydrogenase alpha 1	-2.06	0.0144
BOD1L	biorientation of chromosomes in cell division 1-like	-2.06	0.0150
C3orf23	chromosome 3 open reading frame 23	-2.06	0.0182
TIMM8A	translocase of inner mitochondrial membrane 8 homolog A	-2.06	0.0340
TNPO3	transportin 3	-2.06	0.0142
DNAJC2	DnaJ homolog, subfamily C, member 2	-2.05	0.0191
AKIRIN1	akirin 1	-2.05	0.0234
ARL2BP	ADP-ribosylation factor-like 2 binding protein	-2.05	0.0216
SPAG16	sperm associated antigen 16	-2.04	0.0182
CLCN3	Chloride channel 3	-2.03	0.0153
DEK	DEK oncogene	-2.03	0.0361
RAP2A	RAP2A, member of RAS oncogene family	-2.03	0.0264
RALB	v-ral simian leukemia viral oncogene homolog B	-2.02	0.0114
C1GALT1	core 1 synthase, glycoprotein-N-acetylgalactosamine 3-beta-galactosyltransferase, 1	-2.02	0.0407
GAR1	GAR1 ribonucleoprotein homolog	-2.01	0.0114
NLE1	notchless homolog 1	-2.01	0.0114
CCDC59	coiled-coil domain containing 59	-2.01	0.0114
ROCK2	Rho-associated, coiled-coil containing protein kinase 2	-2.01	0.0296
METTL2A	methyltransferase like 2A	-2.01	0.0144
GBP3	guanylate binding protein 3	-2.01	0.0177
SLC35D1	solute carrier family 35, member D1	-2.01	0.0225
UCHL3	ubiquitin carboxyl-terminal esterase L3	-2.01	0.0386
ATPAF1	ATP synthase mitochondrial F1 complex assembly factor 1	-2.01	0.0153
TMEM9B	TMEM9 domain family, member B	-2.01	0.0113
HNRNPA0	heterogeneous nuclear ribonucleoprotein A0	-2.01	0.0413
USP14	ubiquitin specific peptidase 14	-2.01	0.0309
