# Supplementary material for: Possible Role of miR-375-3p in Cervical Lymph Node Metastasis of Oral Squamous Cell Carcinoma
Source: Cancers (Basel). 2024 Apr 13;16(8):1492. doi: 10.3390/cancers16081492 (PMC11049256; doi:10.3390/cancers16081492)
Supplement: Supplementary file 1 [file cancers-16-01492-s001.zip › Table S6.pdf]

Table S6. Target gene candidates of miR-375-3p in human oral squamous cell carcinoma cells.

Gene symbol	Gene name	Fold change	*P*-value
QKI	quaking homolog, KH domain RNA binding	-9.93	0.0114
MTDH	metadherin	-6.55	0.0202
YAP1	Yes-associated protein 1	-5.54	0.0114
CBX3	chromobox homolog 3	-4.91	0.0119
CHSY1	chondroitin sulfate synthase 1	-4.84	0.0105
MAPKAP1	mitogen-activated protein kinase-associated protein 1	-4.51	0.0140
LDHB	lactate dehydrogenase B	-4.24	0.0142
UBE2E2	ubiquitin-conjugating enzyme E2E2	-4.17	0.0105
USP1	ubiquitin-specific peptidase 1	-3.81	0.0226
C10orf26	chromosome 10 open reading frame 26	-3.72	0.0114
LSM12	LSM12 homolog	-3.57	0.0119
PLEKHA3	pleckstrin homology domain containing, family A member 3	-3.48	0.0105
EBPL	emopamil binding protein-like	-3.42	0.0142
OGFOD1	2-oxoglutarate and iron-dependent oxygenase domain containing 1	-3.18	0.0114
CORO2A	coronin, actin binding protein, 2A	-3.08	0.0245
PTPMT1	protein tyrosine phosphatase, mitochondrial 1	-2.93	0.0181

CSTF2	cleavage stimulation factor, 3’ pre-RNA, subunit 2, 64kDa	-2.92	0.0156
POC1B	POC1 centriolar protein homolog B	-2.89	0.0122
EIF4G3	eukaryotic translation initiation factor 4 gamma, 3	-2.88	0.0105
PPPDE2	PPPDE peptidase domain containing 2	-2.76	0.0144
C1QBP	complement component 1, q subcomponent binding protein	-2.68	0.0179
RPN1	ribophorin I	-2.67	0.0286
MBD2	methyl-CpG binding domain protein 2	-2.63	0.0134
DCUN1D4	DCN1, defective in cullin neddylation 1, domain containing 4	-2.55	0.0142
TMEM55A	transmembrane protein 55A	-2.45	0.0114
ARL4C	ADP-ribosylation factor-like 4C	-2.40	0.0114
PRDX1	peroxiredoxin 1	-2.30	0.0144
KIAA1524	KIAA1524	-2.25	0.0344
MOBKL1B	MOB1, Mps One Binder kinase activator-like 1B	-2.21	0.0114
CNN3	calponin 3, acidic	-2.19	0.0344
ASF1A	ASF1 anti-silencing function 1 homolog A	-2.13	0.0250
PDGFC	platelet-derived growth factor C	-2.12	0.0114
PAQR3	progestin and adipoQ receptor family member III	-2.10	0.0113

CEPT1	choline/ethanolamine phosphotransferase 1	-2.08	0.0262
TIMM8A	translocase of inner mitochondrial membrane 8 homolog A	-2.06	0.0340
DEK	DEK oncogene	-2.03	0.0361
ATPAF1	ATP synthase mitochondrial F1 complex assembly factor 1	-2.01	0.0153
