# Supplementary material for: Possible Role of miR-375-3p in Cervical Lymph Node Metastasis of Oral Squamous Cell Carcinoma
Source: Cancers (Basel). 2024 Apr 13;16(8):1492. doi: 10.3390/cancers16081492 (PMC11049256; doi:10.3390/cancers16081492)
Supplement: Supplementary file 1 [file cancers-16-01492-s001.zip › Table S7.pdf]

Table S7. GSEA in phenotype with miR-375-3p mimic.

Gene sets	NOM *P*-value	FDR *q*-value
GOBP_POSITIVE_REGULATION_OF_GENE_EXPRESSION	0.000	0.060
GOBP_RESPONSE_TO_ABIOTIC_STIMULUS	0.006	0.681
Gender GOBP_RESPONSE_TO_ABIOTIC_STIMULUS	0.006	0.497
GOBP_SMALL_MOLECULE_METABOLIC_PROCESS	0.011	0.710
GOBP_POSITIVE_REGULATION_OF_SIGNALING	0.009	0.848
GOBP_NEGATIVE_REGULATION_OF_PROGRAMMED_CELL_DEATH	0.043	1.000
GOBP_ENDOMEMBRANE_SYSTEM_ORGANIZATION	0.043	1.000
GOBP_TUBE_MORPHOGENESIS	0.046	1.000

GSEA, Gene Set Enrichment Analysis; GOBP, Gene Ontology Biological Process; NOM, Nominal; FDR, False Discovery Rate
